# Neuro-oncology in the Philippines: a scoping review on the state of medical practice, deterrents to care and therapeutic gaps

**DOI:** 10.3332/ecancer.2021.1238

**Published:** 2021-05-20

**Authors:** Mark Willy L Mondia, Adrian I Espiritu, Julette Marie F Batara, Roland Dominic G Jamora

**Affiliations:** 1 Division of Adult Neurology, Department of Neurosciences, College of Medicine and Philippine General Hospital, University of the Philippines Manila, Taft Ave, Ermita, Manila 1000, Philippines; 2 Department of Clinical Epidemiology, College of Medicine, University of the Philippines Manila, Manila 1000, Philippines; 3 Institute for Neurosciences, St. Luke’s Medical Center, Quezon City & Global City 1112, Philippines; a https://orcid.org/0000-0001-8862-5360; b https://orcid.org/0000-0001-5621-1833; c https://orcid.org/0000-0001-5317-7369

**Keywords:** neuro-oncology, treatment gaps, Philippines, healthcare systems

## Abstract

**Background:**

Neoplasms of the brain and spine are relatively uncommon compared to breast, lung and gastrointestinal tumours, which occur at higher rates in the Asian population. Updated guidelines in diagnosis and treatment of neuro-oncologic diseases recommend advanced molecular-based precision-medicine; thus the need for increasingly individualised regimens. It is, therefore, necessary to determine whether there are areas of improvement in the provision of care to these patients, especially in low- to middle-income economies like the Philippines.

**Methods:**

In this study, we identified gaps in the delivery of medical care to Filipino patients with tumours of the central nervous system. We performed a scoping review on the available literature on clinical experience with treatment of neuro-oncologic cases from the Philippines and performed qualitative analysis viewed through the lens of the existing healthcare system.

**Results:**

The medical practice of neuro-oncology in the Philippines lacks robust local data on epidemiology and treatment outcomes. There are existing legislative frameworks to support adequate healthcare delivery and financing to brain tumour patients. However, inequities in the geographic distribution of infrastructure, manpower and medications are roadblocks for accessibility to neuro-oncologic services like specialised molecular markers, neurosurgical procedures, sustained chemotherapy and radiation therapy centres.

**Conclusion:**

There are significant treatment gaps in the care of neuro-oncologic patients in the Philippines that need to be addressed. Early detection and initiation of prognosis-changing therapeutics through reduction of out-of-pocket expenses, access to readily available diagnostic tools and sustainability of management regimens are the main areas that necessitate strengthened partnership between the public and private sectors of Philippine society.

## Introduction

Asia makes up about 60% of the world’s total population, while Southeast Asia (SEA) accounts for 10% of this continent’s population, thus making it a significant contributor in shaping the determinants of health in this part of the world [1]. The Philippines is the second largest country by population size in SEA, and ranks 13th in the world, with a 2020 gross domestic product (GDP) per capita of United States dollar (USD) 3,485.00, classifying it as a lower-middle income country (LMIC) [2, 3].

According to the 2016 report of the World Health Organization (WHO), the global burden of disease for neurological disorders is growing at a disproportionately increasing rate vis-à-vis the capacity of LMICs to cope [3]. Specifically, the latest data reported an increase in age-standardised incidence rates of the central nervous system (CNS) cancer globally by 17.3% as well as 227,000 deaths, which accounted for 7.7 million disability-adjusted life-years (DALYs) between 1990 and 2016 [4]. However, in comparison to lung, colorectal and breast cancer, brain tumours are less common in Asians as well as in the general public [5].

Currently, there is limited published data on the treatment of brain tumours in the Philippines [6]. This discrepancy in relatively low prevalence and high disease burden is hypothesised to be a major impediment for the delivery of holistic care to patients diagnosed with tumours affecting the nervous system. A scoping review approach was applicable due to the lack of extensive systematised knowledge on this potentially practice-changing topic in the local setting. Hence, we aimed to identify and evaluate the treatment gaps in the practice of neuro-oncology in the Philippines.

## Methods

### Protocol

Our study adhered to the Preferred Reporting Items for Systematic reviews and Meta-analyses (PRISMA) guidelines extension for scoping reviews [7].

### Eligibility criteria

We considered published guidelines, meta-analyses, systematic reviews, review articles, randomised controlled trials, prospective/retrospective cohort studies, case series and reports, abstracts, conference proceedings, editorials and textbooks. Human (both paediatric and adult population) and animal studies were included. Articles not in English or Filipino were excluded. No time limitation of publication was set. ‘Neuro-oncology’ was defined as a subject matter dealing with biostatistics, pathogenesis, diagnosis and treatment of brain tumours. Any article published by an author affiliated with an institution in the Philippines not directly dealing with brain tumours was excluded. These were all applied to maximise data sources.

## Information sources

We searched international (i.e. PubMed, Scopus, EMBASE and Clinicaltrials.gov) and local (i.e. Health Research and Development Information Network) medical databases. References of relevant articles were also screened.

We accessed pertinent and available literature via official websites and/or email correspondence with the following: a) international organisations (i.e. World Bank, WHO and PubMed); b) government and non-government agencies (i.e. Philippine Charity Sweepstakes Office (PCSO), Philippine Health Insurance Corporation (PhilHealth), Philippine Amusement and Gaming Corporation, Philippine Cancer Society, Inc. (PCSI), Philippine Alliance of Patient Organizations, Philippine Statistics Authority (PSA), the Office of the President and Department of Health (DOH)); c) medical associations (i.e. Philippine Neurological Association (PNA), Philippine Society of Neuro-Oncology (PSNO), Academy of Filipino Neurosurgeons (AFN), Asian Society for Neuro-Oncology (ASNO), Philippine Radiation Oncology Society (PROS), Philippine Society of Medical Oncology (PSMO), Philippine Society of Pediatric Oncologists (PSPO), Computed Tomography Magnetic Resonance Imaging Society of the Philippines and Philippine Society of Pathologist (PSP)); d) various cancer support groups and e) private medical and pharmaceutical companies.

### Search and selection of sources

We conducted a scoping review of literature starting December 2020–April 2021 using the search term strategy: (central nervous system OR brain OR tumour OR neoplasm) AND (Philippines). MLM and AIE separately searched available literature. All titles and available abstracts were screened based on the eligibility criteria. Duplicates were excluded. We retrieved the full-text of eligible articles for data extraction.

### Data charting process and items

We extracted data regarding epidemiology, legislation, health financing, information systems, workforce, pharmacotherapy and healthcare services in the local treatment of neuro-oncological diseases. Authors, titles and institutional affiliation were extracted from published studies. Lastly, data on distribution, availability and cost of diagnostics and medications were also charted.

### Synthesis of results

Data were mainly qualitatively described and summarised into tables and figures. A conceptual framework was formulated to consolidate the identified treatment gaps.

## Results

### Search of studies

**Figure 1.**
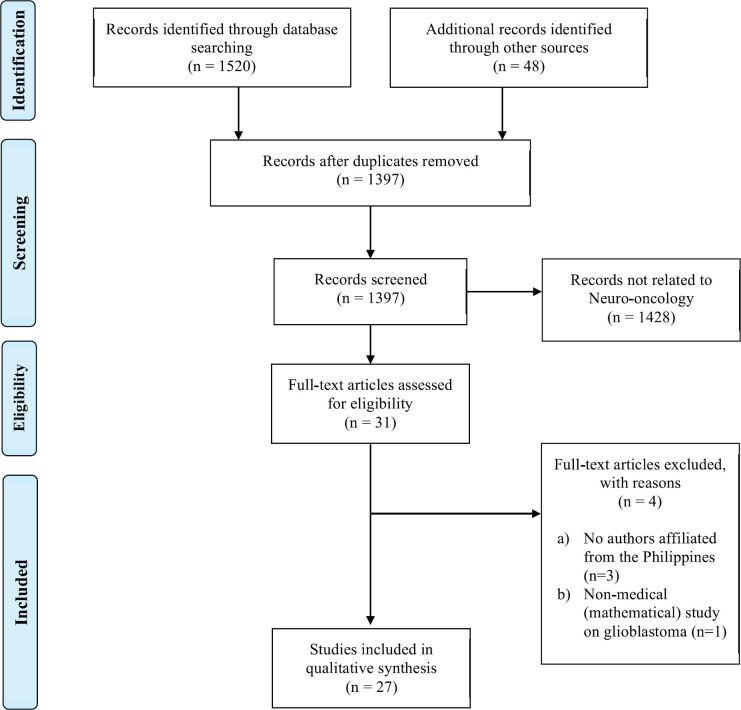
Flow diagram adapted from PRISMA guidelines for scoping reviews.

The search resulted in a total of 1,568 articles (Figure 1). We then screened 1,397 articles after duplicates were removed. We excluded 1,428 articles that were not related to neuro-oncology. Thirty-one full-text articles were assessed for eligibility, where we excluded three articles with no Filipino authors and one mathematical article [8]. Thus, a total of 27 data sources were included for synthesis.

### Local epidemiology of nervous system tumours

A meta-analysis in 2014 noted a world age-standardised incidence rate range for all primary brain tumours to be 4.3–18.6 per 100,000 per year [9]. Internationally, there were 296,851 new cases and 241,037 deaths related to primary nervous system tumours in 2018 [10]. Metastatic brain disease occurs in 10%–15% of patients with cancer and is the most common type of neoplasm in the brain [5]. Based on the latest data in 2019, the global incidence of malignant brain tumours was 4.25 cases per 100,000 person-years, which also varied according to country income group wherein incidence in LMICs was higher at 4.81 [11].

In the Philippines, the burden of disease data in 2016 reported the following: a) incidence of 2,297 cases; b) 1,969 deaths and c) 82,021 DALYs [4]. Based on a 2015 study that looked at the patient profile and outcomes of 262 cases of adult brain tumours from the Philippine General Hospital (PGH), the biggest tertiary hospital in the Philippines, majority of patients were female with a mean age of 41.7 years and a mean symptom duration of 13.2 months [6]. Meningioma, astrocytoma and glioblastoma (GBM) were the most common types [6]. Treatment-wise, 56.5% had surgery, 13.1% had combination therapy and 17.6% had no intervention due to poor prognosis and no patient consent [6]. However, there is a deficiency on updated and extensive local epidemiologic data on neuro-oncological diseases. Data from this 2017 retrospective review from our institution was the only published data regarding CNS tumour incidence in the Philippines. There is no existing national registry programme for CNS tumours in the Philippines.

### Neuro-oncology research in the Philippines

The literature search only yielded one worldwide study tackling gaps in access to care for paediatric CNS tumours, which underscored the lack of data on outcomes, particularly from LMICs [12]. There are no published data specific to the treatment gaps involving adult CNS tumours.

As of April 2021, there are only 14 indexed research outputs from the Philippines dealing primarily with neuro-oncology (Table 1). The remaining articles included for synthesis are references necessary to elaborate on the state of neurologic care for patients with brain tumours in the Philippines. The majority of the articles are case reports. Nine of the twelve studies were published within the last decade with most authors affiliated primarily with PGH. Research productivity for neuro-oncology in the Philippines is still low. There is a need to increase efforts to boost scientific output in this area of neurologic care.

### Philippine healthcare situation

Geographically, the Philippines is an archipelago composed of three main island groups with a total land area of around 300,000 km^2^ and has a population of 110 million as of 2019 [1, 13].

The provision of healthcare in the Philippines is achieved via the public and private sectors. The public sector is directly under the DOH as the main government agency that handles national health policies and services [13]. Particulars of healthcare delivery and policy implementation are under the jurisdiction of a city, municipal and provincial governments [13]. The institutionalisation of universal health is a constant agenda of the national government, but still has limitations in terms of total coverage of costs.

The life expectancy of Filipinos has increased owing to improvements in living conditions and better healthcare provision and access [13]. However, the incidence of noncommunicable diseases particularly diseases of the cardiovascular system and malignant neoplasms have been increasing due to risk factors of diet, tobacco smoke and uncontrolled hypertension [13].

### Philippine health financing

The 2019 Current Health Expenditure (CHE) was 16.37 billion USD (USD 1.00 = PHP 48.40 as of 24 October 2020). The total health expenditure accounted for 87.5% of the CHE and 4.6% of the GDP [14]. However, household out-of-pocket (OOP) payment remained the largest source of health financing comprising 47.9% of CHE, while government schemes and compulsory contributory healthcare financing schemes contributed to 42% of CHE [14]. According to the 2018 PSA data, the average annual family income of Filipinos was USD 6,466.94, with an expenditure of USD 4,938.01 wherein only 2%–3% was used for health, which then leaves only USD 1,549.58 for savings [15].

OOP fees at the point of services via the public sector still contribute a significant chunk of health financing in the Philippines even with efforts to introduce social health insurance since 1995 in the form of PhilHealth [13]. Despite the agency’s high coverage at 92%, its share on the total health expenditure remains relatively low at an average of 30%, thus offering limited financial protection for Filipino households [13].

Legislation pertinent to the practice of medicine and equitable delivery of healthcare, especially towards the vulnerable population in the Philippines, is presented in Supplementary Table 1 [16, 17]. Despite efforts to improve health service delivery, socioeconomic disparities, accessibility and availability of resources, and policy implementation challenges pose threats to ensuring adequate health financing in the Philippines [13].

### Healthcare insurance coverage for neoplasms of the CNS

Public sector insurance via PhilHealth coverage for CNS neoplasms was last updated in 2017. Case rates are divided either into benign or malignant cases. Benign neoplasm of the CNS has a total coverage of USD 304.00 (USD 90.60 for professional fee and USD 213.00 for an institutional fee), while malignant tumours ensure a rate of USD 348.00 (USD 104.00 for professional fee and USD 243.60 for an institutional fee) [18]. Only linear accelerator radiation is covered amounting to USD 61.80 per session [19]. There is no separate dedicated case rate for chemotherapy. Patients usually need to go to other government and non-government organisations for financial assistance for chemotherapy. There are special packages for breast, colon, rectal and prostate cancers depending on the stage that ranges from USD 2,058.00 to 8,235.00, but none for CNS malignancies [19].

### Diagnostics

Based on the 2016 WHO criteria for classifying CNS tumours, both histologic and molecular profiles are necessary [5]. Cranial and spinal imaging are done either via computerised tomography (CT) scan or magnetic resonance imaging (MRI). Data from DOH states that they licensed 429 CT machines (4.2 machines per 1 million population) and 78 MRI units (0.8 MRI units per 1 million population) from 2012 to 2016 used by commercially-owned and government hospitals as well as private imaging facilities mostly found in areas near the highly populated parts of the country [13]. Price ranges from USD 103.00 to 618.00 depending on the area to be imaged as well as the use of contrast [20]. Neurosurgical procedures particularly craniotomy and biopsy with or without excision of tumour have rates from USD 2,060.00 to 4,120.00 [18]. Systemic serum tumour markers are at times included in the work-up for patients suspected with metastasis to the CNS. These tests are usually available in most hospitals and would cost around USD 164.00–247.00 [20]. Histopathologic diagnosis would focus on microscopic analysis using haematoxylin-eosin staining. Immunohistochemistry would utilise stains like synaptophysin, glial fibrillary acidic protein, p53, isocitrate dehydrogenase (IDH) mutation, anti-CD 20, adenosine triphosphate (ATP)-dependent helicase X-linked, KI-67, epithelial membrane antigen [5]. Most secondary and tertiary hospitals would offer these on packages together with the reader's fee of a pathologist and would have prices ranging from USD 62.00 to 617.00 depending if the sample was sent to a public government hospital or private institutions [21, 22]. IDH status and 1p/19p co-deletion are important diagnostics characteristics especially in the classification of low-grade gliomas [5]. Only IDH1 immunostain is available in the country, which is not able to detect IDH2 mutations, hence the need for further gene sequencing. Sequencing for all mutations of the IDH gene as well as O^6^-methylguanine-DNA-methyltransferase methylation status, which is needed for responsiveness to treatment to alkylating agents like temozolomide, is not available in the Philippines and are sent out to the nearest diagnostic centre with such capabilities, usually Singapore. Chromosomal analysis of 1p/19q co-deletion has been recently available at a cost of USD 535.00–1029.00 [21, 23].

### Chemotherapy, radiotherapy and surgical management

According to the National Comprehensive Cancer Network (NCCN) algorithm for the treatment of cancers of the CNS, surgery, chemotherapy and radiotherapy all have roles in treatment^24^. In 2019, the National Integrated Cancer Control Act (NICCA) was created to serve as the framework for all cancer control activities of the Philippine government [16]. The Philippine National Drug Formulary (PNDF) was created and regularly updated, which serves as the basis for cost, procurement and appropriation of essential drugs [25]. Medications included in this formulary are readily available free of charge for admitted patients in government hospitals.

The cost of surgical management would be similar to data in diagnostics since most tumours undergo biopsy or removal of the mass/es either as total or gross total excision. Radiotherapy (RT) is used usually as whole brain radiation therapy, spine RT, partial field RT or stereotactic radiosurgery (SRS) – either concomitantly with chemotherapy in GBMs or chemosensitisers in primary CNS lymphomas, aggressive low grade gliomas or palliation in cases of brain and spine metastasis [24]. As of 2019, there are 50 RT – 33 centres capable of intensity-modulated RT, which is the mainstay of glioma RT regimens; 12 centres capable of performing SRS; but there is only one private hospital in Manila that offers proton beam therapy [26]. Most centres are located in the capital city, Manila, with only 1–2 major hospitals in the other two major island groups (Cebu in Visayas and Davao in Mindanao). Charges for RT usually are based per session and would result in a total of USD 2,000.00–4,000.00 for the treatment duration [6].

Chemotherapy is the backbone of the therapeutic management of malignant CNS tumours [27]. Table 2 shows the common chemotherapeutic drugs used in regimens for brain cancers. Each vial or oral formulation would cost USD 40.00–124.00, wherein one cycle would usually be based on weight or body surface area that would result in USD 1,235.00–3,088.00 with a grand total of USD 4,900.00–30,880.00 for treatment (roughly 4–10 cycles) [28]. Other medications are used for supportive treatment during each cycle, which include corticosteroids, medical decompression, sodium bicarbonate for urine alkalinisation, anti-emetics and leucovorin. Gliomas are usually treated with chemotherapy compared to complete resection for other benign brain tumours. Established treatment with concurrent temozolomide with radiotherapy and subsequent adjuvant temozolomide has been the mainstay treatment for GBM; while high dose methotrexate (HD-MTX) for primary CNS diffuses B cell lymphoma [29, 30]. High-risk low-grade gliomas are either treated with temozolomide or a combination of procarbazine, lomustine and vincristine (PCV) [31]. Access to ancillary tests to check for toxicity especially when using HD-MTX is also an area of concern. Methotrexate assay is only available in two private hospitals in National Capital Region (NCR). Though other hospitals in the capital can send out for this test, administering HD-MTX outside Manila poses a risk of being unable to detect MTX toxicity in a timely manner due to logistical issues.

There are ongoing trials for chemotherapy, immunomodulators, target molecular receptors, vaccines and tumour treating fields. However, all study centres are located in America or European countries [32, 33]. Although it is the recommendation of the NCCN to enrol patients in clinical trials who have advanced malignant brain tumours, this has not been possible due for Filipino patients primarily due to the lack of trial centres in the country [24].

### A multidisciplinary approach to neuro-oncologic care

As of July 2020, there are 544 active PNA board-certified neurologists, with 521 practicing in the Philippines and 23 practicing abroad [34]. Distribution is skewed towards the urbanised centres, where as high as 45% of neurologists practice in the NCR [35]. There are ten accredited neurology training institutions around the country, which offer 3–4 years of residency training as well as fellowship programmes [36]. Currently, there is only one Neuro-oncology fellowship being offered in St. Luke’s Medical Center in Quezon City, Philippines, where the PSNO, a member organisation of the ASNO, is also based [37]. There are only six neuro-oncologists in the country, most of whom had residency training in Adult Neurology and are practicing in hospitals around NCR [38]. For the past decade, there were only two neuro-oncologists in the whole country. This played a significant factor in conducting clinical research and other human resource-related limitations in the practice of this subspeciality in the Philippines.

According to the latest data of the AFN last 2019, there are 131 accredited neurosurgeons in the Philippines, one with a surgical neuro-oncology background and another neurosurgeon trained in medical neuro-oncology [39]. According to the 2019 data of PROS, there are 90 radio-oncologists who practice in 48 radiation therapy facilities in the Philippines [26]. In terms of diagnostic medicine, there are around 500 registered members of the PSP, 3 of whom have fellowships in neuropathology [40]. The ratio of specialists to patient load for the treatment of brain tumors is low. There are only 0.47 neurologists, 0.2 neurosurgeons, and 0.081 radio-oncologists per 100,000 population. As for general medical oncology, there are 284 adult medical oncologists and 35 paediatric oncologists who are recognised by PSMO and PSPO, respectively [41, 42]. In terms of rehabilitation services, there is no particular centre dedicated to brain tumours. However, according to the Philippine Academy of Rehabilitation Medicine as of 2020, there are 452 centres with 316 licensed rehabilitation physicians – most are located in NCR and most of which are privately owned [35].

Neuro-oncology practice in the Philippines is still young compared to more established subspecialities like stroke and epilepsy. The PSNO, as the main society for neuro-oncology in the country, holds biennial conventions and delivers lectures in the PNA and AFN. Through the PGH Department of Neurosciences and PSNO, the first virtual lay forum on brain tumours was held last October 2020 to help spread awareness and education about both benign and malignant CNS tumours. The PNA has yearly research grants and has awarded an ongoing prospective study looking into the quality of life of post-surgical meningioma patients.

### Support services

Due to the high burden of OOP spending for medical care of cancer patients, most Filipinos afflicted with neuro-oncologic diseases would need financial assistance. Government support is available from the Medical Assistance Programme and Individual Medical Assistance Programme of the DOH and PCSO, respectively. These programmes cover provision for confinement, chemotherapy, dialysis and medications limited to haemophilia and post-transplant patients [43]. Since the passing of the ‘*Malasakit*’ (Concern) Center Act of 2019 (Republic Act 11463), such programmes have been centralised to streamline efforts to for patients needing financial help.

There are also non-government organisations that are focused on providing an array of services to cancer patients: PCSI, Amuma Cancer Support Group Foundation, Inc., Cancer Institute Foundation, Inc., Cancer Warriors Foundation, Philippine Brain Tumor Alliance, Philippine Breast Cancer Network, Touched by Max, Inc. and Union for International Cancer Control: Philippines [44]. Particularly, the Philippine Brain Tumor Alliance was founded last 2006 by a patient with GBM and is geared towards raising awareness, information dissemination and empowerment of brain tumour patients [45]. Let’s Save the Brain Foundation (LSTB), an organisation providing aid for the neurologic care of indigent Filipino patients of the PGH Department of Neurosciences, can also be tapped in the provision of diagnostics and chemotherapy medication for neuro-oncology patients [46].

PCSO is the main organisation that patients who need financial assistance for chemotherapy apply to. They would need a protocol and letter from their attending physician. Only one application per patient protocol is allowed in 1 month. Usual cases needing financial aid for chemotherapy are CNS lymphomas and gliomas. HD-MTX and rituximab would cost USD 1,652.89–2,066.11 per cycle every 2 weeks, while temozolomide for GBM would be USD 5,578.51–6,198.35 per cycle in a month. Lomustine as the most potentially most potent part of the PCV regimen for low-grade gliomas is usually imported and is not covered by PCSO. Based on our experience, PCSO would only cover USD 400.00–1,000.00 per month per patient protocol depending on their yearly budget. The remaining balance will be covered by the patient as OOP.

Non-government organisations do not have a pre-set amount that they donate for brain tumour patients. In the case of the LSTB, the application is on a case-to-case basis. They cover from ventricular shunts, cranial imaging for monetary aid, while they only rely on outsourced donations for chemotherapeutic drugs.

### Current practice patterns

In the Philippines, the trajectory of a patient with a brain tumour would depend on the aggressiveness of the disease and the financial burden the patient can tolerate. Since most CNS tumours will either present as headache or seizures, they will be first seen by a general neurologist where cranial imaging will be done revealing a possible tumour. This would cost around USD 300.00–360.00 OOP as an outpatient consult. Another point of contact is when patients come to the emergency department with acute signs of increased intracranial pressure necessitating intensive care admission until a diagnosis of a brain tumour is made. If the patient is admitted to a government hospital, coverage is usually 100%. However, it would cost the patient roughly USD 6,000.00–10,000.00 in the private setting, 80% of which is OOP. In our experience practicing in the country’s biggest government tertiary hospital, we receive around 3–5 cases of brain tumour patients from private hospitals needing further treatment due to depleted funds either prior or after surgery, which can cost up to USD 20,000.00–30,000.00.

In terms of surgical management, most of the big private hospitals (seven in NCR, one in Visayas, two in Mindanao) and tertiary government hospitals have operating microscopes, cortical mapping using functional neuro-imaging for awake craniotomy, can perform stereotactic biopsy and are capable of endoscopic procedures. Patients coming from rural areas would usually need to travel to the locations of these centres to gain access to neurosurgical care, which is an added expense. The main deterrent would be the expense when surgery is done in the private setting. PhilHealth would only cover 20% of the procedure rate and professional fee. Depending on the insurance coverage, most non-emergency neurosurgical cases for brain tumours are not covered. This would still leave an OOP expense of USD 12,000.00. In contrast to public hospitals with neurosurgical services, the cost is fully covered. In terms of more benign tumours like meningiomas, the cost is usually minimal since they will only need to pay for surgical fees and would require no further treatment.

Most of the dilemma is encountered is in the availability of molecular diagnosis. Gene sequencing of IDH1/2 mutation, genetic profiling, O[6]-methylguanine-DNA methyltransferase (MGMT) methylation status are not available, which affect the decision for chemotherapy. RT is usually well-covered by government and private insurances. Methotrexate, rituximab, procarbazine and vincristine are part of the PNDF; while lomustine is not available in the country. The use of formulary drugs can be free if done only on an in-patient basis in government hospitals. However, the main limitation is the number of available stock. For instance, in a HD-MTX infusion every 2 weeks for CNS lymphoma, the patient would need roughly 150 bottles of 50 mg vials, which immediately depletes the hospital’s inventory. Temozolomide regimen for GBM is even more costly. Assistance from PCSO will only cover USD 400.00 out of the USD 5,578.51 per cycle of temozolomide. Overall, the biggest cost in the OOP expense will be coming from the cost of chemotherapy for malignant tumour.

As for benign tumours, treatment is usually adequately achieved with surgery. Pituitary tumours are also well controlled with a combined surgical and medical approach with the collaborative efforts of Neurosurgery, Otorhinolaryngology, Ophthalmology, Neurology and Endocrinology. Thus, most Filipino patients with pineal region tumors receive adequate treatment since the cost for chemotherapy is less expensive compared to CNS lymphomas or gliomas.

## Discussion

Neuro-oncology is constantly faced with practice-changing data due to recent advances in the diagnosis and treatment of tumours of the nervous system [5]. In the Philippines, updated research for brain tumours is lacking. Data on local experience with the management of such cases is also limited. Though there are statutes in place to control the burden of disease of cancer, there are still systemic issues that pose a challenge to timely diagnosis and access to affordable treatment for patients with CNS tumours.

Brain tumours will usually present with seizures, headaches and focal deficits, but can also be indolent; thus making early detection elusive at times [5]. The diagnosis of neuro-oncologic disease relies heavily on histopathology and molecular characterisation, which are relatively costly particularly in the health system of the Philippines wherein in most cases such tests are not covered by government or private insurance. Coupled with urban concertation of most secondary and tertiary hospitals capable of inpatient chemotherapy administration and radiotherapy, complete regimens for control and palliation of CNS tumours are less accessible to a greater majority of citizens of lower socioeconomic strata.

**Figure 2.**
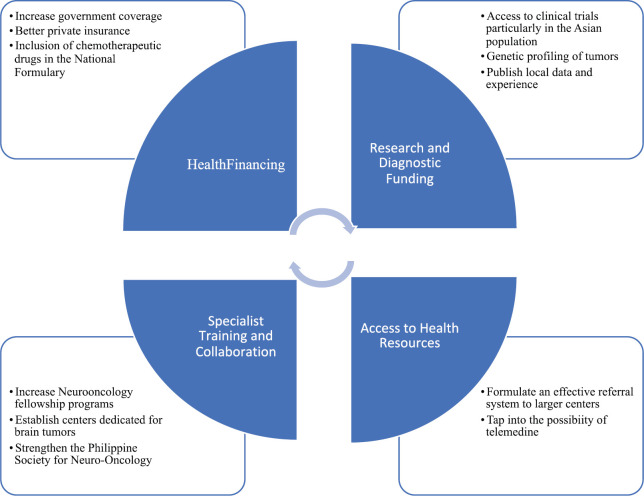
Conceptual framework highlighting the treatment gaps in neuro-oncology in the Philippines.

Earlier studies on Parkinson’s disease, multiple sclerosis and epilepsy identify health financing and proper allocation and availability of therapeutics as gaps in treatment for these neurologic diseases in the Philippines [35, 47, 48]. Neuro-oncologic diseases may share the same issues, but differs significantly in terms of prognosis – malignant CNS neoplasms have dismal survival rates if diagnosis and treatment are delayed [9]. Therefore, it is of utmost importance to implement and fortify systemic changes in legislature and underscore the necessity of a multi-disciplinary team approach to ameliorate the quality of life of these patients. See Figure 2 for our proposed core challenges in the current state of neuro-oncology practice in the Philippines. Also included are some recommendations on how to address these gaps in treatment. From our review and qualitative analysis, health financing and manpower are the main areas of improvement to effectively treat Filipino patients with brain tumours. Physical access to a hospital that can initially diagnose a brain tumour through cranial imaging is a minor issue. The hurdle starts with the upfront cost of neurosurgical procedures for biopsy and/or tumour excision. This can be addressed by lobbying for higher coverage from government insurance as well as urge private insurance companies to cover non-emergency cases for brain surgery. Due to the wide use of RT in almost all cancers, patients are usually able to undergo radiotherapy with full coverage from the government. The second roadblock would be the expensive chemotherapeutic regimens. Patients rely heavily on government financial aid that barely covers half of the total expense of one cycle. A solution to this is to push for non-formulary drugs like temozolomide and lomustine to be available in government hospitals in the appropriate amounts. Currently, an application for temozolomide is being submitted. Lastly, formalising the practice of neuro-oncology in the Philippines by training more neurology and neurosurgical graduates through more fellowship programmes is key to address the lack of subspecialists in this field.

## Conclusion

The published data on incidence and treatment outcomes of CNS tumours in Filipino patients is scarce. Treatment gaps in care for patients with neuro-oncologic diseases include availability and affordability of diagnostics and equitable distribution of facilities and human resources. The main issue with the current practice of neuro-oncology in the Philippines is accessibility to available treatment due to inadequate public and private health insurance coverage of OOP expenses. While efforts towards universal healthcare coverage are a primary thrust of the Philippine health system, there are still areas for improvement especially in addressing the needs of patients with brain tumours. The practice of neuro-oncology can therefore be improved by promoting public awareness, increasing government financial subsidy through appropriate budget allocation specifically by lobbying for creation of specific treatment packages through the existing health financing strategies (i.e. PhilHealth) and generating local epidemiologic and clinical data on benign and malignant neoplasms of the brain.

## List of abbreviations

AFN, Academy of Filipino Neurosurgeons; ASNO, Asian Society for Neuro-oncology; CHE, Current Health Expenditure; CNS, Central nervous system; CT, Computed tomography; DALY, Disability-adjusted life-years; DOH, Department of Health; GDP, Gross domestic product; GBM, Glioblastoma; HD-MTX, High dose methotrexate; IDH, Isocitrate dehydrogenase; LMIC, Lower-middle income country; LSTB, Let’s Save the Brain Foundation; MRI, Magnetic resonance imaging; NCCN, National Comprehensive Cancer Network; NICCA, National Integrated Cancer Control Act; NCR, National capital region; OOP, Out-of-pocket; PCSI, Philippine Cancer Society, Inc.; PCSO, Philippine Charity Sweepstakes Office; PGH, Philippine General Hospital; PhilHealth, Philippine Health Insurance Corporation; PNA, Philippine Neurological Association; PNDF, Philippine National Drug Formulary; PRISMA, Preferred Reporting Items for Systematic Reviews and Meta-Analysis; PROS, Philippine Radiation Oncology Society; PSA, Philippine Statistics Authority; PSMO, Philippine Society of Medical Oncology; PSNO, Philippine Society of Neuro-oncology; PSP, Philippine Society of Pathologist; PSPO, Philippine Society of Pediatric Oncologists; RT, Radiotherapy; SEA, Southeast Asia; USD, United States dollar; WHO, World Health Organization.

## Conflict of interest

The author(s) declare that they have no conflict of interest.

## Financial conflict of interest

The author(s) declare that they have no financial conflict of interest.

## Non-financial conflict of interest

The author(s) declare that they do not have any non-financial conflict of interest.

## Research support/funding

This research did not receive any specific grant from funding agencies in the public, commercial or not-for-profit sectors.

## Competing interests

None.

## Disclosure of results at a meeting

This paper has not been presented in any meeting.

## Institutional review

Not needed.

## Figures and Tables

**Supplementary Table 1.** Pivotal Philippine laws pertinent to the delivery of neuro-oncologic healthcare services.

Law	Name	Description
Republic Act No. 11215	NICCA	Strengthen cancer control in the country, increase cancer survivorship and reduce burden on families and cancer patients
Republic Act No. 11223	Universal Health Care Act of 2019	Provision to every Filipino of the highest possible quality of health care that is accessible, efficient, equitably distributed, adequately funded, fairly financed and appropriately used by an informed and empowered public
Republic Act No. 9502	Universally Accessible Cheaper and Quality Medicines Act of 2008	An act providing for cheaper and quality medicines, amending for the purpose Republic Act No. 8293 or the Intellectual Property Code, Republic Act No. 6675 or the Generics Act of 1988, and Republic Act No. 5921 or the Pharmacy Law, and for other purposes
Republic Act No. 11463	Malasakit Center Act of 2019	Aims to provide medical and financial assistance through a one-stop shop, ‘Malasakit centre’ in all DOH hospitals in the country
Republic Act 9442	Magna Carta for Disabled Persons and for Other Purposes	All persons with disability shall be automatically covered under the National Health Insurance of PhilHealth
Republic Act 9257	Senior Citizen Act	An act granting additional benefits and privileges to senior citizens amending for the purpose Republic Act No. 7432, otherwise known as ‘An Act to Maximise the Contribution of Senior Citizens to Nation Building, Grant Benefits and Special Privileges and for Other Purposes’

**Table 1.** Indexed neuro-oncologic research outputs from the Philippines.

Author	Affiliation	Journal	Title	Description
Dizon-Ocampo and Lovina [49]	Not available	*Annales Paediatrici*	Brain tumors in Filipino children	Retrospective review of primary brain tumours in Filipino children
Billote *et al* [50]	Not available	*Philippine Journal of Pediatrics*	Intracranial teratoma – report of a case	Case report on a paediatric patient
Tan and Rajasoorya *et al* [51]	Fatima College of Medicine Valenzuela, Philippines	*Pituitary*	Metamorphosis of a non-functioning pituitary adenoma to Cushing's disease	Case report
Barrera *et al* [52]	Philippine General Hospital Manila, Philippines	*BMJ Case Reports*	Giant sellar meningioma mimicking pituitary macroadenoma	Case report
Hasselblatt *et al* [53]	St. Luke’s Medical Center Quezon City, Philippines	*Acta Neuropathologica*	Poorly differentiated chordoma with SMARCB1/INI1 loss: a distinct molecular entity with dismal prognosis	Case report
de Roxas *et al* [6]	Philippine General Hospital Manila, Philippines	*Journal of Neurology and Neurorehabilitation Research*	Current treatment status of adult brain tumors in the Philippine general hospital	Retrospective review
Cruz *et. al* [54]	Philippine General Hospital Manila, Philippines	*CNS Oncology*	Holocord oligodendroglioma with intracranial extension in a young adult: a case report and review of literature	Case report
delos Reyes *et al* [55]	University of the East Ramon Magsaysay Memorial Medical Center Quezon City, Philippines	*The Malaysian Journal of Pathology*	Mature teratoma of the pineal region in the paediatric age group: a case report and review of the literature	Case report
Bell *et al* [11]	Philippine General Hospital Manila, Philippines	*Journal of Clinical Neuroscience*	Global incidence of brain and spinal tumors by geographic region and income level based on cancer registry data	Review
Pascual *et al* [56]	Philippine General Hospital Manila, Philippines	*World Neurosurgery*	Awake craniotomy in low-resource settings: findings from a retrospective cohort in the Philippines	Retrospective review for local experience with awake craniotomy
Mondia *et al* [57]	Philippine General Hospital Manila, Philippines	*Frontiers in Oncology*	Primary brain tumor research productivity in Southeast Asia and its association with socioeconomic determinants and burden of disease	Bibliometric study on the research productivity in Southeast Asia (SEA) regarding primary brain tumours, which included research output data from the Philippines
Alonto *et al* [58]	Philippine General Hospital Manila, Philippines	*Current Problems in Cancer*	Rare case of intramedullary spinal cord metastasis from nasopharyngeal carcinoma	Case report
Ignacio *et al* [59]	Philippine General Hospital Manila, Philippines	*Neurological Sciences*	Efficacy of aspirin for sporadic vestibular schwannoma: a meta-analysis	Meta-analysis on treatment
Espiritu *et al* [60]	Philippine General Hospital Manila, Philippines	*World Neurosurgery*	Congenital gliobalstoma multiforme with long-term childhood survival: a case repot and systematic review	Case Report

**Table 2.** Chemotherapeutic drugs used in CNS tumours.

Drug	Formulary medication	Available in the Philippines
Methotrexate 3.5–8 g/m^2^ IV	Yes	Yes
Temozolomide 75–200 mg/m^2^ orally	No	Yes
Rituximab 375 mg/m^2^ IV	Yes	Yes
Lomustine 100–130 mg/m^2^ orally	No	No
Bevacizumab 10 mg/kg IV	No	Yes
Procarbazine 60–00 mg/m^2^	No	Yes
Vincristine 1.4 mg/m^2^ IV	Yes	Yes
Carboplatin 350–560 mg/m^2^ IV	Yes	Yes
Cisplatin 25–100 mg/m^2^/day IV	Yes	Yes
Carmustine 150–200 mg/m^2^ IV	No	Yes
Teniposide 50 mg/m^2^ IV	No	Yes
Irinotecan 340–350 mg/m^2^ IV	No	Yes
Fotemustine 75 mg/m^2^ IV	No	Yes
Cyclophosphamide 750 mg/m^2^ IV	Yes	Yes
Etoposide 100 mg/m^2^/day IV	Yes	Yes
Cytarabine 2–3 g/m^2^ IV	Yes	Yes
Ifosfamide 1.5 g/m^2^ IV	Yes	Yes
Capecitabine 1,000–2,400 mg/m^2^/day orally	Yes	Yes
Lapatinib 1,250 mg orally	No	Yes
Dabrafenib 150 mg orally	No	Yes
Vemurafenib 960 mg orally	No	Yes
Topotecan 1.5 mg/m^2^ IV	No	Yes
